# An epigenetic classifier for early stage lung cancer

**DOI:** 10.1186/s13148-018-0502-3

**Published:** 2018-05-22

**Authors:** Yun Su, Hong Bin Fang, Feng Jiang

**Affiliations:** 1 Department of Surgery, Jiangsu Province Hospital of Nanjing University of Chinese Medicine, 138 Xianlin Road, Nanjing, 210023 China; 2 Department of Biostatistics, Bioinformatics and Biomathematics, Georgetown University Medical Center, 4000 Reservoir Road, N.W, Washington D.C., 20057 USA; 3 Department of Pathology, University of Maryland School of Medicine, Baltimore, MD USA

**Keywords:** ddPCR, DNA methylation, Sputum, Diagnosis, Lung cancer

## Abstract

**Background:**

Methylated genes detected in sputum are promise biomarkers for lung cancer. Yet the current PCR technologies for quantification of DNA methylation and diagnostic value of the sputum biomarkers are not sufficient to be used for lung cancer early detection. The emerging droplet digital PCR (ddPCR) is a straightforward means for precise, direct, and absolute quantification of nucleic acids. Here, we investigate whether ddPCR can sensitively and robustly quantify DNA methylation in sputum for more precise diagnosis of lung cancer.

**Results:**

First, the analytic performance of methylation-specific ddPCR (ddMSP) and quantitative methylation-specific PCR (qMSP) is determined in methylated and unmethylated DNA samples. Second, 29 genes, previously proposed as potential sputum biomarkers for lung cancer, are analyzed by using ddMSP in a training set of 127 lung cancer patients and 159 controls. ddMSP has higher sensitivity, precision, and reproducibility for quantification of methylation compared with qMSP (all *p* < 0.05). A classifier comprising four sputum methylation biomarkers for lung cancer is developed by using ddMSP, producing 86.6% sensitivity and 90.6% specificity, independent of stage and histology of lung cancer (all *p* > 0.05). The classifier has higher accuracy compared with sputum cytology (88.8 vs. 70.6%, *p* < 0.01). The diagnostic performance is confirmed in a testing set of 89 cases and 107 controls.

**Conclusions:**

ddMSP is a robust tool for reliable quantification of DNA methylation in sputum, and the epigenetic classifier could help diagnose lung cancer at the early stage.

**Electronic supplementary material:**

The online version of this article (10.1186/s13148-018-0502-3) contains supplementary material, which is available to authorized users.

## Background

Lung cancer is the leading cause of cancer death among men and women [1]. More than 85% lung tumors are non-small cell lung cancers (NSCLCs), which consist of adenocarcinoma (AC), squamous cell carcinoma (SCC), and large cell carcinoma (LC). Cigarette smoking is the foremost cause of NSCLC [2]. People who smoke cigarettes are nearly 30 times more likely to get lung cancer or die from lung cancer than people who do not smoke. Even smoking a few cigarettes a day or smoking occasionally increases the risk of lung cancer. Individuals who quit smoking have a lower risk of lung cancer than if they had continued to smoke, but their risk is higher than the risk for people who never smoked. The National Lung Screening Trial (NLST) results show that using low-dose CT (LDCT) for the early detection of lung cancer in smokers can reduce the mortality by 20% as compared to chest X-rays [1]. Therefore, LDCT is recently recommended to be used for lung cancer early detection among smokers [3, 4]. However, LDCT is associated with over-diagnosis, excessive cost, and radiation exposure, limiting its clinical applications [3–5]. The development of noninvasive approaches that can accurately and cost-effectively diagnose early stage lung cancer among smokers remains clinically important [6].

Lung cancer develops from a field defect characterized by an accumulation of molecular abnormalities resulted from repeated exposure of the airway of the smokers to the tobacco-related carcinogens [7–9]. Regardless of the anatomic location relative to the tumors, the molecular alterations observed in the large bronchial airway might reflect the altered changes existed in lung tumors [9–11]. Sputum is defined as secretions from the airways and contains bronchial epithelial cells exfoliated from the airways or lungs [12]. Therefore, the analysis of exfoliated bronchial epitheliums in sputum for the molecular changes may provide a useful tool for noninvasively and cost-effectively diagnosing lung cancer.

DNA methylations of tumor suppressor genes (TSGs) are early molecular events in lung carcinogenesis and thus show great promise as biomarkers for early stage lung cancer [6, 11, 13–34]. Conventional qPCR-based platforms, particularly, methylation-specific PCR (qMSP), have been used for detecting DNA methylation of TSGs in sputum [13]. However, qMSP has some weaknesses, limiting its use in the clinical settings. For example, qMSP is an indirect approach, which requires internal controls for data normalization [35]. Furthermore, qMSP’s sensitivity for analyzing low copy number of genes is poor. This is particularly challenging for quantification of DNA methylation in bronchial epitheliums, as the large excess of non-epithelial cells in sputum could obscure detection of the relative scarcity of methylated DNA from the exfoliated bronchial epitheliums. A more sensitive, precise, and reproducibility method for quantification of methylated DNA in sputum would provide a useful means for noninvasive diagnosis of lung cancer.

Droplet digital PCR (ddPCR) is a direct method for quantitatively measuring nucleic acids [36–45], since it depends on limiting partition of the PCR volume, where a positive result of a large number of microreactions indicates the presence of a single molecule in a given reaction. The number of positive reactions, together with Poisson’s distribution, can be used to produce a straight and high-confidence measurement of the original target concentration [43]. Furthermore, ddPCR does not require the reliance on rate-based measurements, endogenous controls, and the use of calibration curves. In addition, previous studies including our own research have demonstrated that ddPCR can quantify low-abundance nucleic acids and has higher sensitivity and precision than does conventional PCR [36, 37, 46]. The objective of this study is to investigate whether methylation-specific ddPCR (ddMSP) could sensitively and robustly quantify DNA methylations in sputum and hence develop a biomarker-based classifier for early stage lung cancer.

## Methods

### Study population

The study protocol was approved by the local Institutional Review Board. The participants in this study were recruited from the hospital at the point of their referral for suspected lung cancer between the ages of 55–80. Written informed consent was obtained from all enrolled subjects. Exclusion criteria included pregnancy, current pulmonary infection, surgery within 6 months, radiotherapy within 1 year, and life expectancy of < 1 year. Clinical diagnosis of lung cancer was made using histopathologic examinations of specimens obtained by CT-guided transthoracic needle biopsy, transbronchial biopsy, videotape-assisted thoracoscopic surgery, or surgical resection. The surgical pathologic staging was determined according to the TNM classification of the International Union Against Cancer with the 8th American Joint Committee on Cancer and the International Staging System for Lung Cancer. Histopathological classification was determined according to the World Health Organization classification. A total of 482 subjects including 216 lung cancer patients and 266 cancer-free smokers were recruited. The 216 lung cancer patients were diagnosed with NSCLC consisting of 55 stage I cases, 55 stage II cases, 50 stage III cases, and 56 stage IV cases. One hundred and twelve cases were AC, 91 were SCC, and 13 were LC. The 266 cancer-free patients who were smokers and served as control subjects had granulomatous inflammation (*n* = 117), nonspecific inflammatory changes (*n* = 105) or lung infections (*n* = 44). The cancer-free smokers had been followed for at least 2 years, and none had any evidence of cancer. No difference of age, gender, and smoking status was observed in the lung cancer cases vs. controls (All *p* > 0.05). To refine the biomarkers whose changes specific to NSCLC, the cases were matched to the controls on gender, age, race, and smoking status as a nested case-control study. The cases and controls were then randomly split into a training set and a testing set by using a random number generator. The training set consisted of 127 lung cancer patients and 159 cancer-free controls. The testing set comprised 89 lung cancer patients and 107 cancer-free controls. The demographic and clinical characteristics of the two cohorts are presented in Tables 1 and 2.

**Table 1:** Characteristics of NSCLC patients and cancer-free smokers in a training set

	NSCLC cases (*n* = 127)	Controls (*n* = 159)	*p* value
Age	65.48 (SD 12.32)	65.63 (SD 11.56)	0.32
Sex			0.35
Female	45	56	
Male	82	103	
Smoking pack-years (median)	35.23	33.79	0.34
Stage			
Stage I	33		
Stage II	32		
Stage III	29		
Stage IV	33		
Histological type			
Adenocarcinoma	63		
Squamous cell carcinoma	57		
Large cell carcinoma	7		

*Abbreviations*: *NSCLC* non-small cell lung cancer

**Table 2:** Characteristics of NSCLC patients and cancer-free smokers in a testing set

	NSCLC cases (*n* = 89)	Controls (*n* = 107)	*p* value
Age	65.25 (SD 11.28)	65.36 (SD 11.48)	0.30
Sex			0.36
Female	31	37	
Male	58	70	
Smoking pack-years (median)	35.76	33.29	0.32
Stage			
Stage I	22		
Stage II	23		
Stage III	21		
Stage IV	23		
Histological type			
Adenocarcinoma	49		
Squamous cell carcinoma	34		
Large cell carcinoma	6		

*Abbreviations*: *NSCLC* non-small cell lung cancer

### Sample collection and sputum cytology

Sputum was collected from the participants as described in previous reports [47–54]. Briefly, to reduce the percentage of oral epithelial cells in the sputum, subjects were asked to blow their nose, rinse their mouth, and swallow water to minimize contamination of squamous cells from postnasal drip and saliva. Sputum samples were then coughed in a sterile container and processed within 2 h. To further minimize oral squamous cell contamination, opaque or dense portions that looked different from saliva under the inverted microscope were selected using blunt forceps from expectorate. The samples were processed on ice in 4 volumes of 0.1% dithiothreitol (Sigma-Aldrich, St. Louis, Mo) followed by 4 volumes of phosphate-buffered saline (PBS) (Sigma-Aldrich). The cell suspension was filtered through 45-μm nylon gauzes (BNSH Thompson, Scarborough, ON, Canada). Absolute cell numbers and cell viability were quantitated by using a hemacytometer with trypan blue. Two cytocentrifuge slides were prepared from aliquots of cell suspension by using a cytospin machine (Shandon, Pittsburgh, PA) and were then stained with the Papanicolaou staining technique [12]. A sputum sample was considered adequate if lung macrophages or Curschmann spirals were present on the slides [11, 12]. Cytologic diagnosis was performed on the cytospin slides using the classification of Saccomanno et al. [12]. The remaining cells are stored at − 80 °C until used.

### DNA isolation and bisulfite conversion

We extracted DNA from the specimens using DNeasy kit (Qiagen, Valencia, CA) as previously described [14]. We eluted DNA with 50 μL of elution buffer (10 mmol/L Tris-Cl, pH 8.5) (Sigma-Aldrich Corporation). DNA was quantified by using the Quantifiler Human DNA Quantification kit (Applied Biosystems, Foster City, CA). Bisulfite conversion was carried out on DNA by using the Zymo EZ DNA Methylation Kit (Zymo Research, Irvine, CA) according to the manufacturer’s protocol.

### Serially diluted methylated/unmethylated DNA specimens

We purchased 100% methylated and 100% unmethylated control human DNA samples (Zymo Research). We isolated DNA from sputum of a healthy nonsmoker whose sputum DNA did not harbor DNA methylation of TSGs, including *RASSF1A*, *3OST2*, and *PRDM14* [14]. To determine limit of quantification (LOQ) of an assay, we diluted methylated DNA into the sputum DNA sample in the following concentrations: 100, 25, 6.25, 1.56, 0.39, 0.1, 0.04, and 0% methylated DNA. To determine limits of detection (LOD) of an assay, we prepared serially diluted samples containing 5000, 2500, 1250, 625, 313, 156, and 0 pg methylated DNA in H2O.

### Quantification of DNA methylation in sputum by ddMSP

We added bisulfite-treated DNA (2 μL) to ddPCR mixture (18 μL) containing 2 × ddPCR Supermix for probes (no-dUTP), 750 nmol/L of each primer and 250 nmol/L of the corresponding probe in a final volume of 20 μL. Twenty-nine genes were selected for DNA methylation analysis, since the genes were previously reported as potential sputum methylation biomarkers for lung cancer [6, 13–34]. The 29 genes are *3OST2*, *APC*, *CDH1*, *CDO1*, *CXCL*, *CYGB*, *DAL-1*, *DAPK*, *DCR2*, *FAM19A4*, *FHIT*, *GATA*, *H-cadherin*, *HOXA9*, *JPH3*, *KIFLA*, *MAGE*, *p16*, *PAX5*, *PCDH20*, *PHACTR3*, *PRDM14*, *RARβ*, *RASSF1A*, *SOX17*, *SULF2*, *TAC1*, *TCF2L*, and *ZFP42* (Additional file 1: Table S1). Primers and probes of the targeted genes were designed in the studies [6, 13–34]. A thermocycling protocol (95 °C × 10 min; 40 cycles of [94 °C × 30s, 60 °C × 60s], 98 °C × 10 min) was undertaken in a Bio-Rad C1000 (Bio-Rad, Pleasanton, CA). The PCR plate was transferred to the QX100 Droplet Reader (Bio-Rad) for automatic reading of samples in all wells. We used QuantaSoft 1.7.4 analysis software (Bio-Rad) and Poisson statistics to compute droplet concentrations (copies/μL; PCR scale). Only tests that had at least 10,000 droplets were used for the ddMSP analysis [36, 37]. All assays were done in triplicates, and one no-template control and two interplate controls were carried along in each experiment.

### Quantification of DNA methylation in sputum by qMSP

qMSP was done as previously described [13, 14]. The cycle threshold (Ct) values for each gene were determined. Ct values above 35 were censored according to previous recommendations [13, 14, 55–58]. To determine methylation level of target genes in a given sample, we normalized Ct values of the target genes in relation to that the of *myoblast determination protein one* (*MYOD1*) [13, 32]. The percentage of methylated reference (PMR) was defined as target gene*/MYOD1* ratio of the sample divided by target gene*/MYOD1* ratio of the calibrator DNA (methylated control DNA) and multiplying by 100 [14].

### Comparison of tolerance of ddMSP and qMSP to PCR inhibitors

To determine tolerance of ddMSP and qMSP to inhibitory substances of PCR, we directly introduced inhibitors, sodium dodecyl sulfate (SDS), and heparin (Sigma-Aldrich Corporation), into the PCR reactions [59, 60]. Differences in the resulting inhibition curves and the half-maximal inhibitory concentrations (IC50) were assessed and compared as described previously [59, 60].

### Statistical analysis

We used *t* test to determine significant differences of values of each gene between cases and controls. We used log transformation of the molecular results and applied Pearson’s correlation analysis to assess relationship between DNA methylation and demographic characteristics of subjects. We calculated coefficient of variations (CV) to determine the variation between different measurements. We performed the linear regression between different measurements of the assays and the amount of input DNA. We used the receiver-operator characteristic (ROC) curve and area under the curve (AUC) to determine accuracy, sensitivity, and specificity of each gene or the tests. We employed logistic regression models with constrained parameters as in least absolute shrinkage and selection operator (LASSO) based on ROC criterion to eliminate the irrelevant genes and optimize a composite biomarker panel (classifier). The optimal panel of biomarkers was blindly applied to the testing data set to confirm the diagnostic value by comparing the AUC with the goodness-of-fit statistics [61].

## Results

### ddMSP has higher sensitivity, precision, and reproducibility for quantification of DNA methylation compared with qMSP

**Fig. 1:**
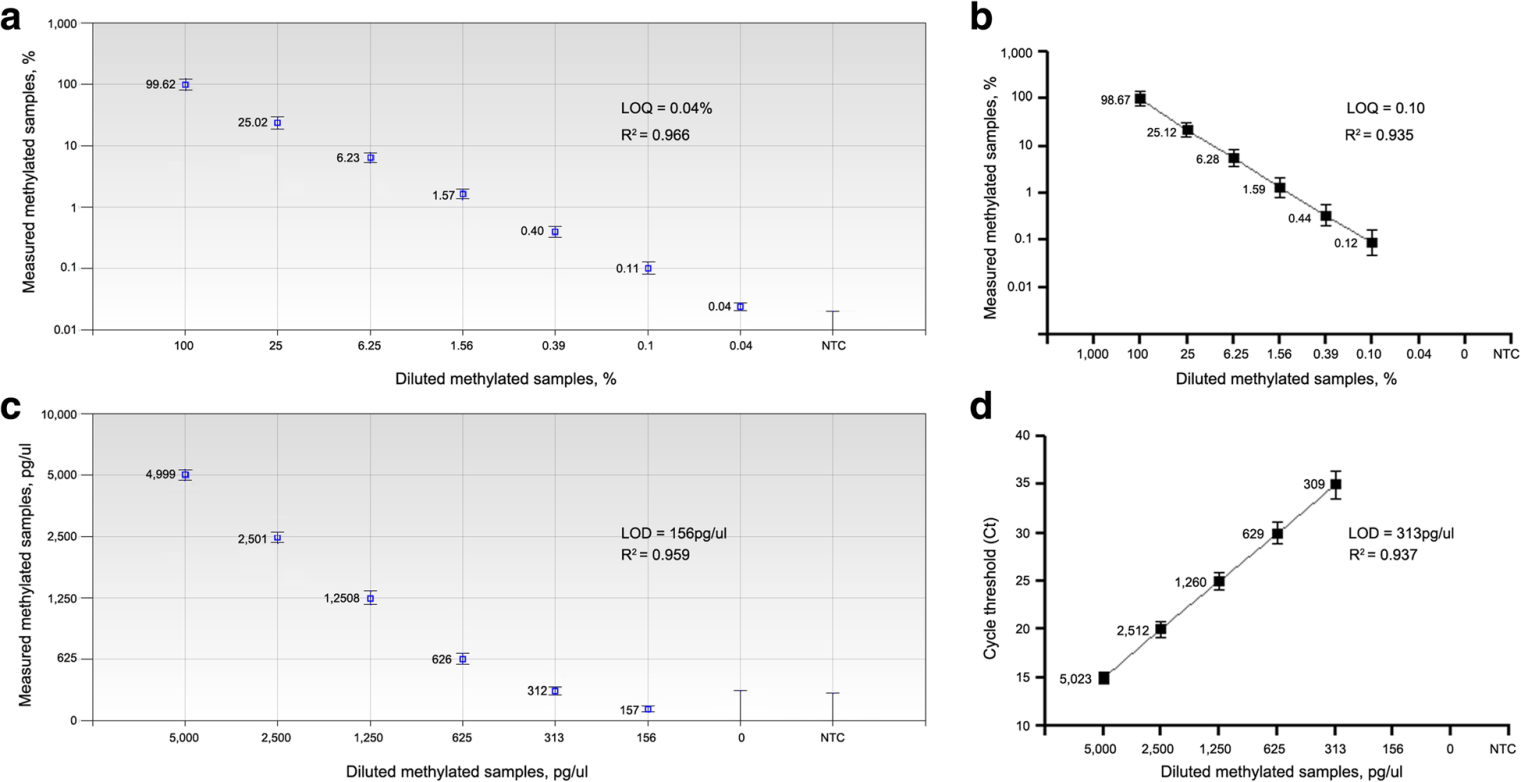
The dynamic ranges and sensitivities of ddMSP and qMSP for quantification of DNA methylation. **a** In methylated DNA serially diluted in sputum DNA of a healthy nonsmoker, ddMSP can detect levels of methylated *3OST2* as low as 0.04% (LOQ = 0.04%). A negative template control (NTC) sample was also tested. *R*^2^ = 0.966 shows excellent linear correlation between measured concentration of methylated DNA and expected percentage of methylation. **b** qMSP can detect methylated *3OST2* at 0.10% (LOQ = 0.10%) in the same diluted samples with *R*^2^ of 0.935. **c** In 100% methylated DNA serially diluted into water, ddMSP can detect the smallest amount of methylated DNA at 156 pg/μL (156 pg/μL) with *R*^2^ of 0.959. **d** qMSP can detect the smallest amount of methylated DNA at 156 pg/μL (156 pg/μL) with R2 of 0.937

In methylated DNA serially diluted in sputum DNA of a healthy nonsmoker, ddMSP generated at least 10,000 droplets passing through a fluorescence detector. The results suggested that the specimens were successfully “read” by ddPCR. ddMSP detected methylated genes (*RASSF1A*, *3OST2*, and *PRDM14*) at a concentration of 0.04% (LOQ = 0.04%)(*R*^2^ = 0.966) (Fig. 1a), whereas qMSP detected the methylation at a concentration of 0.10% (LOQ = 0.10%)(*R*^2^ = 0.935) (*p* = 0.008) (Fig. 1b). There was excellent linearity between the methylated DNA input and values measured by both qMSP and ddMSP (all *R*^2^ ≥ 0.93). Furthermore, the dispersion of values of the four analyses of the specimen was lower with ddMSP than with qMSP. The repeated measurements by ddMSP had a lower CV value compared with those determined by qMSP (*p* = 0.03) (Additional file 1: Table S2). Therefore, ddMSP had a higher precision for quantification of methylation compared to qMSP (*p* = 0.03) (Additional file 1: Table S2).

To determine the absolute LOD of the two platforms, 100% methylated DNA serially diluted into water and then tested by ddMSP and qMSP. The smallest amount of methylated DNA that can be reliably measured by ddMSP was 156 pg/μL (Fig. 1c), suggesting that ddMSP had a LOD of 156 pg/μL. qMSP produced more than 35 Ct values for the samples that had less than 313 pg methylated DNA per microliter, yielding a LOD of 313 pg/μL (Fig. 1d). Therefore, ddMSP had higher sensitivity as demonstrated by lower LOQ and LOD than did qMSP in the serial dilutions of DNA control samples (all *p* < 0.001).

To determine reproducibility of ddMSP and qMSP, the diluted samples were independently analyzed. The CVs of repeated measures by ddMSP on different days were more than twofold lower compared with those determined by qMSP (Additional file 1: Table S3). Furthermore, the CVs of repeated measures by different research staff using ddMSP were at least twofold lower than did those generated by qMSP (Additional file 1: Table S4). Therefore, ddMSP had a higher reproducibility than did qMSP for quantification of DNA methylation.

**Fig. 2:**
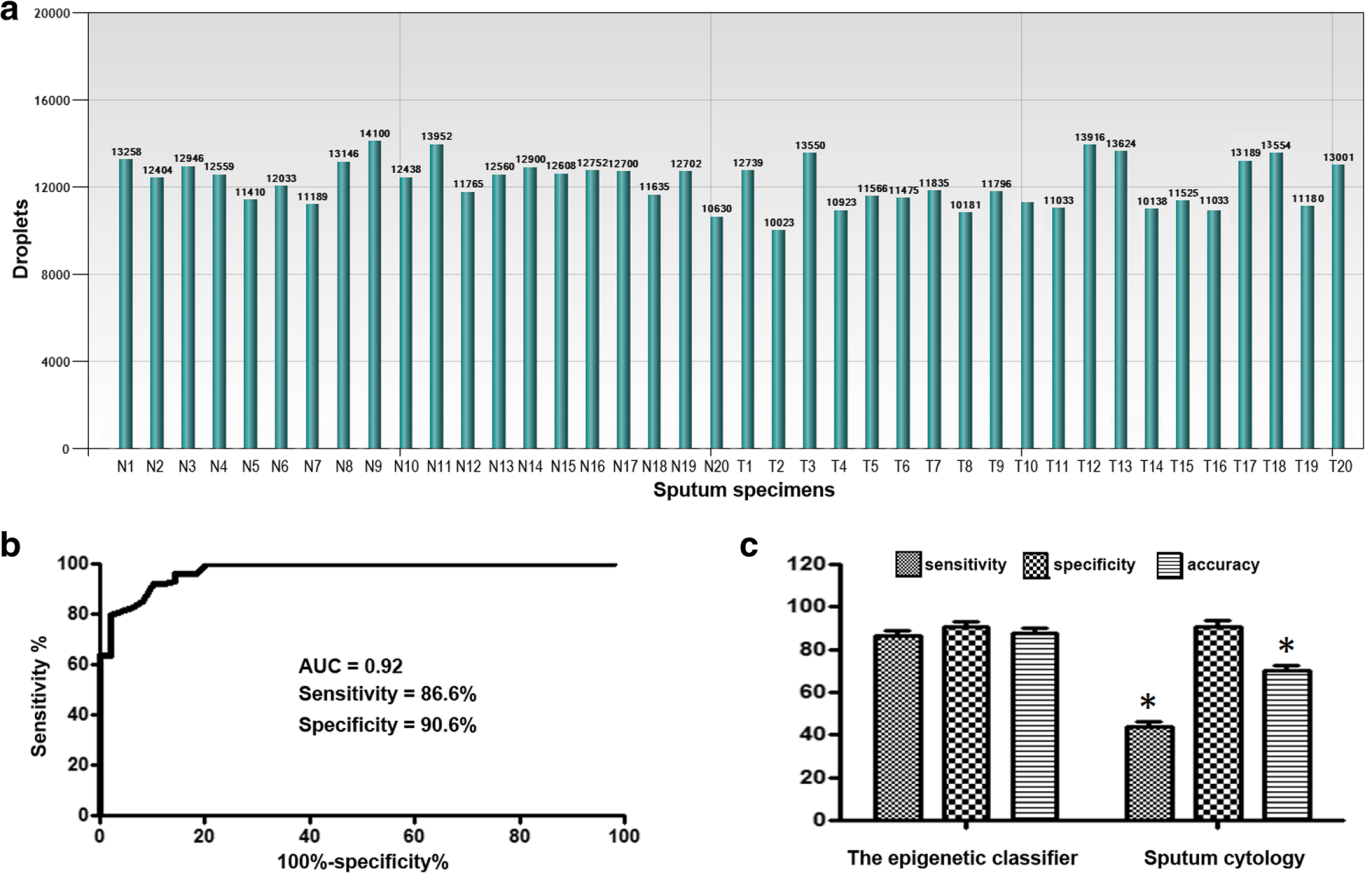
ddMSP and qMSP analyses of DNA methylation in clinical sputum samples. **a** ddMSP analysis of sputum samples of 20 cancer-free controls (normal subjects, *N*) and 20 patients diagnosed with lung tumor (T) for DNA methylation of *RASSF1A*. Each well of the sputum samples contained at least 10,000 droplets, suggesting that the clinical sputum specimens could be successfully “read” by ddPCR. **b** ddMSP analysis of 29 genes in a training set of 127 lung cancer patients and 159 controls developed an epigenetic classifier consisting of four DNA methylation biomarkers. The epigenetic classifier produced 0.92 AUC with 86.6% sensitivity and 90.6% specificity for diagnosis of lung cancer. **c** The epigenetic classifier had higher accuracy (88.8 vs. 70.63%, *p* = 0.004) and sensitivity (86.6 vs. 44.8%, *p* < 0.001) compared with sputum cytology, whereas keeping a similar specificity (90.6 vs. 91.2%, *p* = 0.34)

To evaluate analytic performance of ddMSP and qMSP in clinical sputum samples, sputum of 20 lung cancer patients and 20 cancer-free controls was tested for *RASSF1A*, whose aberrant methylation level was shown to be elevated in sputum of lung cancer patients [13, 14, 55–58]. Each well of the samples contained at least 10,000 droplets (Fig. 2a). Therefore, ddMSP analysis of DNA methylation could successfully be performed in clinical sputum specimens. *RASSF1A* analyzed by both the techniques displayed a high methylation level in lung cancer patients vs. controls (all *p* < 0.05). In the ddMSP assay, a specimen with ≥ one copy of DNA methylation of *RASSF1A*per microliter was considered to be positive. When the criteria was used, of 20 sputum specimens of lung cancer patients, 11 (55%) had positive methylation of the gene detected by ddMSP. In the qMSP assay, a PMR ≥ 1% was classified as positive for *RASSF1A* in a given sample [62]. When the criteria was used, 9 (45%) were positive for *RASSF1A* by qMSP. The same 4 sputum specimens of control subjects had positive methylation of the gene detected by both ddMSP and qMSP. Therefore, ddMSP analysis of DNA methylation of *RASSF1A* in sputum had a higher sensitivity (55%) than did qMSP (45%) (*p* = 0.01) for distinguishing lung cancer patients from control subjects, while maintaining the same specificity (80%) (Additional file 1: Figure S1). Furthermore, the CVs of repeated measures by ddMSP on different days by different researchers were approximately twofold lower compared with those generated by qMSP. Altogether, in clinical sputum specimens, ddMSP also exhibited higher sensitivity, accuracy, and reproducibility than did qMSP for quantification of DNA methylation.

To compare the tolerance of ddMSP and qMSP to PCR inhibitors, we added SDS and heparin directly into the PCR reactions and then calculated log IC50 values from the resulting inhibition curves. We found greater than a half log increase in IC50 of ddMSP over qMSP for both SDS and heparin (all *p* < 0.05), implying that ddMSP tolerated the presence of the inhibitors better than qMSP.

### Diagnostic performance of ddMSP quantified-sputum methylation biomarkers for lung cancer

We first evaluated DNA methylation of 29 genes in the training cohort of 127 NSCLC patients and 159 controls. All the 29 genes displayed a higher level of methylation in patients vs. controls (all *p* < 0.05). ROC curve and AUC analysis showed that the genes had 29–88% sensitivities and 26–92% specificities in differentiating lung cancer patients from healthy controls (Additional file 1: Table S1). Since methylation levels of genes did not follow a normal distribution, we used the log transformation of ddPCR results. We then applied multivariate logistic regression models with stepwise regression based on ROC curve to develop a prediction classifier. Four genes (*HOXA9*, *RASSF1A*, *SOX17*, and *TAC1*) were identified as the best biomarkers (all *p* < 0.001) and incorporated into a logistic classifier: Probability of lung cancer = *e*^*x*^/(1 + *e*^*x*^), where *x* = 1.69 + 1.48 × log (*HOXA9*) − 1.25 × log (*RASSF1A*) + 0.27 × log (*SOX17*) + 0.16 × log (*TAC1*). The logistic classifier produced 0.92 AUC for lung cancer detection (Fig. 2b). Furthermore, Pearson correlation among methylation levels of the four genes was low (*p* > 0.05), implying that their diagnostic values were complementary to each other. Using Youden’s index, we set up optimal cutoff at 1.28 for the prediction classifier. Subsequently, combined use of the four genes by simply calculating the equation produced 86.6% sensitivity and 90.6% specificity. In addition, including other genes in the prediction classifier did not improve the accuracy for lung cancer diagnosis. The prevalence of the DNA methylation of the four genes was related with pack-years of smoking (*p* = 0.03). Since the cases and controls were matched 1:1 by age, gender, and smoking status as a nested case-control study, we adjusted the parameters during model building. The logistic classifier did not show special association with stage and histological type of lung cancer, and patients’ age, gender, and smoking status (all *p* > 0.05). Moreover, the logistic classifier had higher accuracy (88.8 vs. 70.63%, *p* = 0.004) and sensitivity (86.6 vs. 44.8%, *p* < 0.001) than did sputum cytology, while maintaining a similar specificity (90.6 vs. 91.2%, *p* = 0.34) (Fig. 2c). The integrated use of the biomarkers and sputum cytology did not significantly increase the diagnostic value.

### Validating the panel of ddMSP-quantified methylation biomarkers in a testing cohort

In the testing cohort, the panel of the four genes had 85.4% sensitivity and 91.6% specificity in differentiating lung cancer patients from controls (Table 3). In line with findings in the training set, the logistic classifier was not associated with patient’s age, gender, and smoking status, as well as histological type and stage of NSCLC (all *p* > 0.05). Moreover, the logistic classifier yielded higher accuracy (88.8 vs. 71.4%, *p* = 0.002) and sensitivity (85.4 vs. 46.1%, *p* < 0.001), while keeping a similar specificity (91.6 vs. 92.5%, *p* = 0.45), as compared with sputum cytology (Table 3). Taken together, the validation data confirmed the potential of the ddMSP-quantified sputum biomarkers as a sensitive classifier for the early detection of lung cancer.

**Table 3:** The diagnostic performance of the epigenetic classifier and sputum cytology in a testing set

	Accuracy (%) (95% CI)*	Sensitivity (%) (95% CI)*	Specificity (%) (95% CI)
The epigenetic classifier	88.78 (83.50 to 92.83)	85.39 (76.32 to 91.99)	91.59 (84.63 to 96.08)
Sputum cytology	71.43 (64.56 to 77.64)	46.07 (35.44 to 56.96)	92.52 (85.80 to 96.72)

*Abbreviations*: *NSCLC* non-small cell lung cancer, *CI* confidence interval

*p < 0.05

## Discussion

This current study presents the earliest assessment of ddPCR, an emerging technique, for quantitative detection of DNA methylation in sputum. We find that ddMSP can absolutely and robustly quantify DNA methylation in sputum without requiring external references. Therefore, determination of DNA methylation in sputum by ddMSP is highly efficient, and data handling is forthright. Furthermore, our head-to-head comparison of ddMSP and qMSP reveals that ddMSP displays higher precision and reproducibility in measuring copy number of DNA methylation in both control DNA samples and clinical sputum specimens. The sensitivity of conventional qMSP for analyzing low-abundance methylated DNA is poor. This is particularly challenging for the determination of DNA methylation in bronchial epitheliums, since the large excess of normal cells in sputum could obscure detection of the relative scarcity of methylated DNA. We find that ddMSP has a higher sensitivity to quantify cancer-specific methylation and thus could overcome the obstacle of qMSP. In addition, the total time required for ddMSP is about twofold shorter than did qMSP and might be further reduced when an automated system is used [63, 64]. Moreover, ddMSP is not expensive and tolerates the PCR inhibitors better compared with conventional qMSP. Altogether, ddMSP is a straightforward and robust approach for accurate quantification of DNA methylation in sputum.

Importantly, using ddMSP, we develop and validate a DNA methylation-based classifier that has higher accuracy and sensitivity compared with sputum cytology, the clinical gold standard. Furthermore, ddMSP analysis of the four genes by simply calculating the equation would be a convenient tool to be used in the clinics. In addition, the diagnostic performance of the logistic classifier is independent of stage and histological type of the NSCLC, as well as age, gender, and smoking status of subjects. Therefore, the classifier has an important characteristic if it is employed for more precisely and easily identifying early stage lung cancer among smokers.

However, some limitations do exist in this present study: (i) we evaluate ddMSP for quantification of DNA methylation using a retrospective cases and controls, which may produce selection bias and overfitting. Furthermore, the cases and controls are hospital-based patients, and not representative of the smokers in a screening setting for lung cancer early detection. We will perform a large trial to prospectively validate if the logistic classifier could help identify lung cancer at the early stage in a screening setting among smokers. (ii) the overall sensitivity and specificity of the DNA methylation-based classifier are 86.6 and 90.6%, which are not high enough for clinical diagnosis of NSCLC. The integration of the methylation biomarkers with other classes of biomarkers, such as microRNAs [36, 47–52, 65–67] or DNA mutations [33], is one path to improve the early detection of lung cancer [14]. (iii) since there is no sample left from the patients of the training and cohorts, we are not able to test the classification performance of qMSP in all the samples of the training and testing cohorts. We are consenting new cases and controls and collecting specimens and will then compare performance of qMSP and ddMSP in the same samples. (iv) like qMSP, ddMSP has the same limitations as do other PCR-based platforms. For instance, large experiments can become quite labor intensive to perform, when multiple genes are targeted. In this present study, we use 96-well PCR plates. Given the four genes to be analyzed with duplication, we only test 12 clinical samples at one time. In the future, we will use 484-well PCR plates, in which, we could simultaneously test 60 samples.

## Conclusion

ddMSP could be a sensitive and robust tool for reliable quantitation of sputum methylation biomarkers. The ddMSP-quantified methylation classifier may provide a potential diagnostic test for the early detection of lung cancer and thus reduce the deaths and costs associated with the disease. Nevertheless, the continued development of this new technology, and further exploring its value for routine use for the early detection of lung cancer would be required.

## Additional file

Additional file 1: **Table S1.** Twenty-nine genes displayed a higher level of methylation in sputum of lung cancer patients vs. controls. **Table S2.** CVs of the four analyses by ddMSP and qMSP on the same specimens. **Table S3.** CVs of repeated measures by ddMSP and qMSP on different times. **Table S4.** CVs of repeated measures by different researches using ddMSP and qMSP. **Figure S1.** Sensitivities and specificities of measuring DNA methylation of *RASSF1A* by ddMSP and qMSP for diagnosis of lung cancer in sputum samples of 20 cancer-free controls (normal subjects, N) and 20 patients diagnosed with lung tumor (T). ddMSP analysis of DNA methylation of *RASSF1A* had a higher sensitivity (55%) than did qMSP (45%) (*p* = 0.01) for distinguishing lung cancer cases from controls, while maintaining the same specificity (80%). *, *p* = 0.01. (PDF 235 kb)
