# Supplementary material for: An epigenetic classifier for early stage lung cancer
Source: Clin Epigenetics. 2018 May 22;10:68. doi: 10.1186/s13148-018-0502-3 (PMC5964676; doi:10.1186/s13148-018-0502-3)
Supplement: Supplementary file 1 — Table S1. Twenty-nine genes displayed a higher level of methylation in sputum of lung cancer patients vs. controls. Table S2. CVs of the four analyses by ddMSP and qMSP on the same specimens. Table S3. CVs of repeated measures by ddMSP and qMSP on different times. Table S4. CVs of repeated measures by different researches using ddMSP and qMSP. Figure S1. Sensitivities and specificities of measuring DNA methylation of RASSF1A by ddMSP and qMSP for diagnosis of lung cancer in sputum samples of 20 cancer-free controls (normal subjects, N) and 20 patients diagnosed with lung tumor (T). ddMSP analysis of DNA methylation of RASSF1A had a higher sensitivity (55%) than did qMSP (45%) (p = 0.01) for distinguishing lung cancer cases from controls, while maintaining the same specificity (80%). *, p = 0.01. (PDF 235 kb) [file 13148_2018_502_MOESM1_ESM.pdf]

## Supplementary file

**Supplementary table 1. Twenty-nine genes displayed a higher level of methylation in sputum of lung cancer patients vs. controls**

| Genes | Sensitivity | 95% confidence interval | Specificity | 95% confidence interval |
|---|---|---|---|---|
| *3OST2* | 0.53 | 0.46–0.56 | 0.86 | 0.83–0.89 |
| *APC* | 0.44 | 0.34–0.55 | 0.88 | 0.71–0.96 |
| *CDH1* | 0.36 | 0.18–0.57 | 0.71 | 0.52–0.85 |
| *CDO1* | 0.79 | 0.68–0.87 | 0.68 | 0.44–0.86 |
| *CXCL* | 0.37 | 0.27–0.47 | 0.80 | 0.72–0.86 |
| *CYGB* | 0.52 | 0.46–0.58 | 0.80 | 0.68–0.87 |
| *DAB2* | 0.04 | 0.02–0.09 | 0.98 | 0.95–0.99 |
| *DAL-1* | 0.31 | 0.22–0.39 | 0.87 | 0.79–0.92 |
| *DAPK* | 0.46 | 0.41–0.52 | 0.80 | 0.65–0.89 |
| *DCR2* | 0.42 | 0.32–0.51 | 0.61 | 0.53–0.69 |
| *FAM19A4* | 0.81 | 0.73–0.87 | 0.26 | 0.22–0.31 |
| *FHIT* | 0.53 | 0.44–0.62 | 0.92 | 0.78–0.99 |
| *GATA* | 0.67 | 0.32–0.90 | 0.54 | 0.33–0.72 |
| *H-cadherin* | 0.51 | 0.23–0.76 | 0.58 | 0.45–0.69 |
| *HOXA9* | 0.80 | 0.64–0.90 | 0.51 | 0.17–0.85 |
| *JPH3* | 0.32 | 0.22–0.43 | 0.80 | 0.72–0.86 |
| *KIFLA* | 0.45 | 0.36–0.56 | 0.63 | 0.55–0.71 |
| *MAGE* | 0.46 | 0.35–0.57 | 0.83 | 0.57–0.93 |
| *MGMT* | 0.43 | 0.33–0.53 | 0.92 | 0.76–0.97 |
| *p16* | 0.49 | 0.41–0.57 | 0.91 | 0.83–0.95 |
| *PAX5* | 0.38 | 0.28–0.46 | 0.79 | 0.71–0.85 |
| *PCDH20* | 0.59 | 0.49–0.68 | 0.50 | 0.40–0.59 |
| *PHACTR3* | 0.61 | 0.53–0.67 | 0.69 | 0.63–0.74 |
| *PRDM14* | 0.63 | 0.57–0.67 | 0.77 | 0.73–0.81 |
| *RARβ* | 0.45 | 0.29–0.62 | 0.80 | 0.59–0.91 |
| *RASSF1A* | 0.29 | 0.22–0.39 | 0.96 | 0.92–0.96 |
| *SOX17* | 0.85 | 0.76–0.90 | 0.89 | 0.69–0.98 |
| *SULF2* | 0.52 | 0.41–0.62 | 0.58 | 0.49–0.66 |
| *TAC1* | 0.87 | 0.76–0.91 | 0.76 | 0.52–0.90 |
| *TCF2l* | 0.30 | 0.21–0.38 | 0.72 | 0.62–0.79 |
| *ZFP42* | 0.88 | 0.77–0.92 | 0.64 | 0.40–0.82 |

**Supplementary table 2.** CVs of the four analyses by ddMSP and qMSP on the same specimens

| Diluted samples | ddMSP | | | | | | | qMSP | | | | | | | Fold |
|---|---|---|---|---|---|---|---|---|---|---|---|---|---|---|---|
| | Analyse #1 | #2 | #3 | #4 | mean | SD | %CV | Analyse #1 | #2 | #3 | #4 | mean | SD | %CV | |
| 100.00 | 101.00 | 98.00 | 100.00 | 98.00 | 99.00 | 1.41 | 1.43 | 103.00 | 97.00 | 103.00 | 99.00 | 101.00 | 3.00 | 2.99 | 2.09 |
| 25.00 | 23.00 | 25.00 | 26.00 | 25.00 | 25.50 | 0.71 | 2.77 | 22.00 | 26.00 | 25.00 | 23.00 | 24.00 | 1.83 | 7.61 | 2.74 |
| 6.25 | 6.23 | 6.26 | 6.25 | 6.23 | 6.24 | 0.01 | 0.23 | 6.21 | 6.22 | 6.30 | 6.26 | 6.24 | 0.04 | 0.66 | 2.90 |
| 1.56 | 1.57 | 1.54 | 1.57 | 1.56 | 1.57 | 0.01 | 0.45 | 1.60 | 1.54 | 1.57 | 1.53 | 1.56 | 0.03 | 2.03 | 4.49 |
| 0.39 | 0.38 | 0.39 | 0.40 | 0.39 | 0.40 | 0.01 | 1.79 | 0.39 | 0.35 | 0.40 | 0.39 | 0.39 | 0.02 | 5.80 | 3.24 |
| 0.10 | 0.11 | 0.09 | 0.10 | 0.11 | 0.11 | 0.01 | 6.73 | 0.14 | 0.11 | 0.09 | 0.12 | 0.12 | 0.02 | 18.10 | 2.69 |
| 0.04 | 0.03 | 0.02 | 0.04 | 0.03 | 0.04 | 0.01 | 20.20 | | | | | | | | |
| 0.00 | | | | | | | | | | | | | | | |

Cv, coefficient of variation.

**Supplementary table 3.** CVs of of repeated measures by ddMSP and qMSP on different times

| Diluted samples | ddMSP | | | | | | qMSP | | | | | | Fold |
|---|---|---|---|---|---|---|---|---|---|---|---|---|---|
| | Day 1 | 2 | 3 | mean | SD | %CV | Day 1 | 2 | 3 | mean | SD | %CV | |
| 5000.00 | 5001.00 | 5008.00 | 4992.00 | 5001.00 | 6.56 | 0.13 | 5009.00 | 4998.00 | 4972.00 | 4998.00 | 19.00 | 0.38 | 2.90 |
| 2500.00 | 2503.00 | 2506.00 | 2490.00 | 2503.00 | 7.14 | 0.29 | 2512.00 | 2500.00 | 2478.00 | 2500.00 | 17.24 | 0.69 | 2.42 |
| 1250.00 | 1249.00 | 1235.00 | 1253.00 | 1249.00 | 7.90 | 0.63 | 1243.00 | 1228.00 | 1264.00 | 1243.00 | 18.08 | 1.45 | 2.29 |
| 625.00 | 626.00 | 626.00 | 622.00 | 626.00 | 2.00 | 0.32 | 628.00 | 625.00 | 612.00 | 625.00 | 8.50 | 1.37 | 4.28 |
| 313.00 | 312.00 | 317.00 | 310.00 | 312.00 | 2.99 | 0.95 | 310.00 | 320.00 | 306.00 | 310.00 | 7.21 | 2.31 | 2.42 |
| | 154.00 | 149.00 | 156.00 | 154.00 | 2.99 | 1.95 | | | | | | | |
| 0.00 | | | | | | | | | | | | | |

CV, coefficient of variation.

**Supplementary table 4.** CVs of repeated measures by different researches using ddMSP and qMSP

| Diluted samples | ddMSP | | | | | qMSP | | | | | Fold |
|---|---|---|---|---|---|---|---|---|---|---|---|
| | Researcher #1 | Researcher #2 | mean | SD | %CV | Researcher #1 | Researcher #2 | mean | SD | %CV | |
| 5000.00 | 5003.00 | 5008.00 | 5006 | 3.54 | 0.07 | 5010.00 | 4993.00 | 5001.50 | 12.02 | 0.24 | 3.40 |
| 2500.00 | 2505.00 | 2502.00 | 2504 | 2.12 | 0.06 | 2499.00 | 2505.00 | 2502.00 | 4.24 | 0.17 | 2.83 |
| 1250.00 | 1245.00 | 1233.00 | 1239 | 8.49 | 0.48 | 1241.00 | 1220.00 | 1230.50 | 14.85 | 1.21 | 2.49 |
| 625.00 | 627.00 | 623.00 | 625 | 2.83 | 0.32 | 622.00 | 630.00 | 626.00 | 5.66 | 0.90 | 2.82 |
| 313.00 | 311.00 | 316.00 | 314 | 3.54 | 0.80 | 312.00 | 322.00 | 317.00 | 7.07 | 2.23 | 2.80 |
| 156.00 | 153.00 | 158.00 | 156 | 3.54 | 1.61 | | | | | | |
| 0.00 | | | | | | | | | | | |

CV, coefficient of variation.

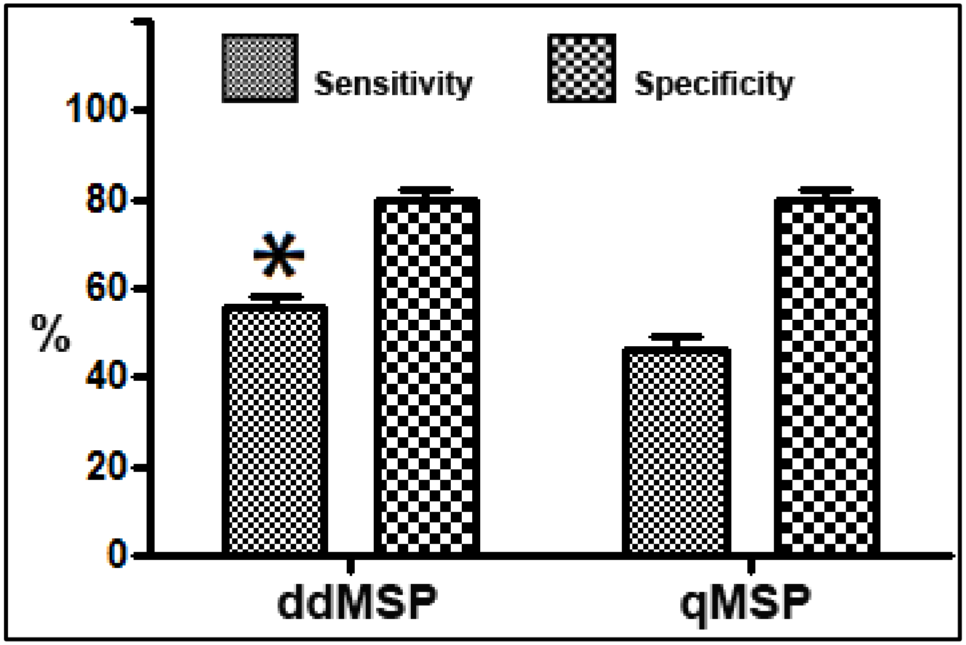


**Supplementary Fig. 1.** Sensitivities and specificities of measuring DNA methylation of RASSF1A by ddMSP and qMSP for diagnosis of lung cancer in sputum samples of 20 cancer-free controls (normal subjects, N) and 20 patients diagnosed with lung tumor (T). ddMSP analysis of DNA methylation of RASSF1A had a higher sensitivity (55%) than did qMSP (45%) ($p=0.01$) for distinguishing lung cancer cases from controls, while maintaining the same specificity (80%). *, $p=0.01$.
